# Leveraging Big Data to Improve Health Awareness Campaigns: A Novel Evaluation of the Great American Smokeout

**DOI:** 10.2196/publichealth.5304

**Published:** 2016-03-31

**Authors:** John W Ayers, J Lee Westmaas, Eric C Leas, Adrian Benton, Yunqi Chen, Mark Dredze, Benjamin M Althouse

**Affiliations:** ^1^ Graduate School of Public Health, San Diego State University Chula Vista, CA United States; ^2^ American Cancer Society Behavioral Research Center Atlanta, GA United States; ^3^ University of California San Diego School of Medicine La Jolla, CA United States; ^4^ Human Language Technology Center of Excellence Johns Hopkins University Baltimore, MD United States; ^5^ Bryn Mawr College Bryn Mawr College Philadelphia, PA United States; ^6^ Santa Fe Institute Santa Fe, NM United States; ^7^ New Mexico State University Las Cruces, NM United States

**Keywords:** big data, evaluation, health communication, mass media, social media, tobacco control, infodemiology, infoveillence, twitter, smoking cessation

## Abstract

**Background:**

Awareness campaigns are ubiquitous, but little is known about their potential effectiveness because traditional evaluations are often unfeasible. For 40 years, the “Great American Smokeout” (GASO) has encouraged media coverage and popular engagement with smoking cessation on the third Thursday of November as the nation’s longest running awareness campaign.

**Objective:**

We proposed a novel evaluation framework for assessing awareness campaigns using the GASO as a case study by observing cessation-related news reports and Twitter postings, and cessation-related help seeking via Google, Wikipedia, and government-sponsored quitlines.

**Methods:**

Time trends (2009-2014) were analyzed using a quasi-experimental design to isolate spikes during the GASO by comparing observed outcomes on the GASO day with the simulated counterfactual had the GASO not occurred.

**Results:**

Cessation-related news typically increased by 61% (95% CI 35-87) and tweets by 13% (95% CI −21 to 48) during the GASO compared with what was expected had the GASO not occurred. Cessation-related Google searches increased by 25% (95% CI 10-40), Wikipedia page visits by 22% (95% CI −26 to 67), and quitline calls by 42% (95% CI 19-64). Cessation-related news media positively coincided with cessation tweets, Internet searches, and Wikipedia visits; for example, a 50% increase in news for any year predicted a 28% (95% CI −2 to 59) increase in tweets for the same year. Increases on the day of the GASO rivaled about two-thirds of a typical New Year’s Day—the day that is assumed to see the greatest increases in cessation-related activity. In practical terms, there were about 61,000 more instances of help seeking on Google, Wikipedia, or quitlines on GASO each year than would normally be expected.

**Conclusions:**

These findings provide actionable intelligence to improve the GASO and model how to rapidly, cost-effectively, and efficiently evaluate hundreds of awareness campaigns, nearly all for the first time.

## Introduction

Public health awareness campaigns are annual events designed to foster knowledge of health risks and engagement during specific days, weeks, or months. The number of public awareness campaigns has substantially risen, with one catalogue listing more than 200 ongoing domestic campaigns [1].

The ubiquity of awareness campaigns likely stems from the ease of their implementation. While most mass media campaigns require substantial advertising budgets [2], awareness campaigns require minimal assets. For example, many awareness campaigns are accomplished by issuing a press release that is freely carried across news media outlets and potentially on social media.

The ease of implementing awareness campaigns, however, sharply contrasts with the pragmatic and methodological barriers that have made assessing their potential effectiveness unfeasible. As an example, a recent systematic review found that only 5 of 79 published reports on awareness campaigns were evaluation studies [3]. One reason for the absence of evaluations is that the driving incentive for awareness campaigns is to minimize operational expenses, which can make traditional evaluations too costly [4]. In addition, because most awareness campaigns occur on a single day, traditional evaluations may not be possible, such as collecting survey responses for a pre-post analysis. Regardless, traditional modalities alone cannot altogether inform how awareness campaigns are expected to work, as they often cannot assess how campaign messages are diffused via news media, are shared on social media, or how the population engages with these messages (such as seeking out information online). Alternative evaluation approaches that rely on metrics assessing both media and popular reactions to media are needed to ensure that awareness campaigns’ important public health messages are scientifically supported.

Big data may fill the knowledge gap for evaluating health awareness campaigns. It has to be noted that other public health surveillance gaps are already being filled by big data’s inexpensive, fine-grained, and real-time capabilities, especially in cases where little or no traditional data exist [5-7]. However, to date, big data have rarely been applied to studying health communication campaigns [8-14], or even health communication research more broadly [15-18]. The few studies harnessing big data have often relied on a single outcome, such as Internet search queries, and loosely interpreted these outcomes in the absence of any framework for campaign evaluations. We take a step forward by proposing a simple and actionable approach to monitoring trends in the most frequently mentioned kinds of big data (news media, social media, and help seeking on Google, Wikipedia, and telephone counseling services). Our analyses are presented in the context of a novel evaluation framework that takes into account how awareness campaigns are expected to diffuse [3], and thus provide actionable intelligence for program managers at each stage of campaign diffusion from news media at the population level to individual help seeking regarding campaign outcomes.

In this report, we specifically developed a framework to evaluate the *American Cancer Society’s* “Great American Smokeout” (GASO). This past year (2015) marked the 40th commemoration of the GASO, held annually on the third Thursday of November, making it the world’s longest running health awareness campaign. The GASO aims to generate and diffuse media encouraging individuals to go smoke-free for a day, and to consider quitting for good. Although activities promoting the GASO have varied over time, GASO is currently promoted by scheduling interviews with selected news outlets; producing media fact sheets with information about tobacco harms and quitting tips; disseminating toolkits with infographics, email templates, and event ideas such as quitting competitions for partnering organizations or businesses to celebrate the GASO; and more recently, by posting similar messages on social media.

Our aim was to develop a method for studying how a message diffuses through news and social media and ultimately leads to engagement via information seeking with the core message. Our novel evaluation framework was based on the key intended aims of the GASO and available big data sources. First, we examined the extent to which the GASO’s cessation-related messages for 2009-2014 were carried in news media; second, how similar messages were shared on Twitter; and third, the extent to which these messages were associated with help seeking via Internet searches for quitting smoking, information retrieval on Wikipedia’s cessation page, and calling state-sponsored quitlines, all informed by an analysis of 2191 days of data. We hypothesized that trends would spike on the day of the GASO. Moreover, the interrelationships between these data (eg, news media’s relationship with social media) were explored, under the assumption that news media begets social media, progressing through individual help-seeking actions for cessation.

## Methods

Data were compiled into a daily time series for the period between January 1, 2009, and December 31, 2014. Each data source was purposively selected to provide insights on a different level of analysis or specificity. For example, news media trends indicate how well GASO’s message of quitting smoking is disseminated, whereas search query trends indicate that the searcher is thinking about cessation or that the searcher is taking some action toward investigating behavior change. Table 1 presents the diverse data sources, as briefly described in the following section.

Cessation-related news media coverage that mentioned “quit” or “stop” and “smoking” was retrieved from News Library, a comprehensive repository of US newspaper articles, including online and print content. This included stories with titles such as “The last cigarette: Nine ex-smokers who quit the habit for good,” “Quit smoking, one of the most important ways to improve health,” and “Quitting smoking reduces heart risk faster than previously thought.” Stories mentioning cessation were normalized relative to all news stories to adjust for potential biases arising from changes in media volume over time.

Social media shares about cessation were obtained from the public Twitter application programming interface. This did not include any data that had been marked as “private” by the author or any direct messages. Initially, the data were derived from 2 feeds, each a random sample of 10% of all tweets. We retained 600,000 tweets mentioning “quit” or “stop” and “smoking” (including variations on “quit” like “quitting”) while not mentioning “fire,” “marijuana,” “mj,” “pot,” “pott,” or “weed.” Subsequently, we randomly selected 10,000 of these tweets and a single reader labeled them as to whether the tweeter was indeed tweeting about smoking cessation. We then trained an automated classifier on that data to mimic human coding, obtaining about 90% precision and 70% recall. The resulting classifier was ran on all 600,000 tweets to obtain the analyzed trend. This included tweets such as “tryn quit smoking waiste to much money” (sic) or “quit smoking and start exercising because healthy body healthy mind” (sic). Tweets were measured in raw volume, but trending (increases in tweets over time) was corrected for in the analysis as detailed in the following section.

Internet searches for cessation information were obtained from *Google Trends*, a public archive of Google search queries. Queries that included “quit” or “stop” and “smoking” (such as “quit smoking methods,” “help stop smoking,” or “quit smoking”) after excluding searches that also included “fire,” “marijuana,” “mj,” “pot,” “pott,” or “weed,” were monitored relative to all queries each day, reported as a relative search volume (RSV), where RSV=100 is the day with the highest search proportion, and RSV=50 is a day with 50% of that highest proportion.

**Table 1:** Big data codebook.

Type	Source	Description	Privacy	Aim
News coverage	News Library [19]: Available with a paid subscription, including papers since 1903.	Includes news articles from 5689 US newspapers. Described as “virtually all” US newspapers.	All publicly available data. Complete text not available for some papers behind pay walls.	To evaluate how frequently the primary GASO message was carried by news media.
Social media	Twitter [20]: Available prospectively with a paid subscription.	Includes either samples of all tweets or all tweets for specific keywords/phrases on the global Twitter platform.	Derived from public tweets (excluding direct messages or tweets marked “private”). Includes username and location for users sharing their location.	To evaluate how frequently cessation-related tweets were shared on social media (a secondary aim of the GASO).
Internet searches	Google [21]: Available to any user with a Google account.	Includes global search trends for investigator-selected keywords or phrases.	Derived from private search activity on Google. Disaggregated to city, national, or global units without any identifying information to protect privacy.	To evaluate how the GASO motivated some to engage in behavior change by seeking out additional information on cessation.
Information retrieval	Wikipedia [22]: Raw access logs are available here; those used in the study were aggregated by a third party.	Includes counts of all Wikipedia page visits. Aggregated by language, but unable to aggregate by location.	Derived from private online activity. Disaggregated to preserve privacy.	To evaluate how the GASO motivated specific information retrieval on the most popular cessation resource.
Quitline calls	Sourced privately, covering 29 US states. Additional states are only available from other providers or state representatives.	Includes call language, frequency, and duration to US state-sponsored quitlines.	Derived from privately obtained calls. Data were aggregated nationally to protect privacy.	To evaluate how the GASO motivated some smokers to engage in quit counseling.

Wikipedia visits to the primary English language “smoking cessation” entry were also counted. Wikipedia was selected because it is typically the first or second result on Internet search engines. Web page views for this Wikipedia page were normalized by dividing by all Wikipedia English language page views reported per 1,000,000 each day.

Quitline call records were obtained from aggregating daily call logs for 28 states whose quitline service is provided by Alere Wellbeing, Inc. Demographic details for each call are recorded (eg, language of the call), but here we focused on all English-language calls. The raw call counts were used because there was no suitable denominator for normalization; however, trending (and other problems normalization typically resolves) was corrected for in the analysis as detailed in the following section.

Our statistical approach was quasi-experimental, focusing on deriving a single treatment effect for any potential spike proximal to the GASO in the time series for each of the 5 data sources [18,23]. The treatment period was the day of the GASO and the reference was based on the empirically derived counterfactual had the GASO not occurred. For the overall estimate we used autoregressive integrated moving average (ARIMA) models that included all the days in each time series with a regression coefficient for the days of GASO fit to maximize Akaike information criterion using the algorithm outlined by Hyndman and Khandakar [24]. For the individual years, we fit the same ARIMA models to the year prior to each GASO event. The counterfactual value for the GASO was imputed based on this model for each of the 5 analyzed time series. The ratio of the observed values (treatment) and derived values from the ARIMAs (counterfactual) was computed, thereby making the effect estimates comparable across each time series [eg, (Treatment − Counterfactual)/Counterfactual]. These models are robust to the most well-known biases. For instance, the models are adjusted for seasonal [25] and circaseptan (day of the week) [26,27] periodicities, and trending in the data, such as declines in smoking-cessation Internet search queries as a proportion of all queries due to shrinking smoking populations or how cessation tweets may be growing as Twitter’s user base grows. Confidence bounds (alpha=.05) around these estimates were estimated using 10,000 random draws from the multivariate normal sampling distribution with mean equal to the maximum-likelihood point estimates, and variance equal to the variance-covariance matrix [28].

To examine how increases in each time series were related to increases in media, we fit a linear model with the percent increases on GASO for media as the predictor and the percent increases for tweets, search, Wikipedia, and quitline calls as the outcome. Coefficients of these models were used to estimate the percent increase in outcome for a given increase in media. Confidence intervals were calculated analogously to those above.

Last, we replicated the aforementioned models focusing on estimating treatment effects for New Year’s Day, which typically sees the greatest spike in cessation-related activity. These estimates were used as a baseline to judge the practical impacts of the GASO relative to New Year’s Day (eg, ΔNYD divided by ΔGASO).

## Results

**Figure 1:**
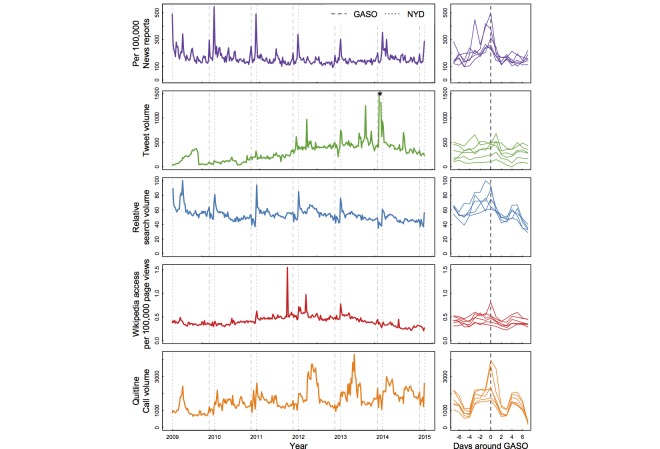
Trends in all outcome measures (news volume, Tweets, Google searches, Wikipedia article views, and quitline call volume, top to bottom panels) across the entire study period (2009-2014). Gray lines indicate GASO (dashed lines) and New Years Day (NYD, dotted lines). The right panels display the same data focused around the days before and after the GASO for each year of the study period.

Three patterns were observed from visual inspection of the time series across outcomes **(** Figure 1). There was substantial variability across outcomes in the size of the spikes (if any) around the GASO. For example, the GASO concurred with large spikes in cessation-related news media in 2009 but it was not replicated in tweets. There was substantial year-to-year variability within outcomes in the size of the spikes around the GASO. For example, news media coverage of cessation appeared to be following a downward trend with potential spikes appearing smaller later in the study period. Last, it appeared that the timing of the GASO closely preceded or occurred during lows in many of the outcomes. For example, Google searches were at their annual low around the holiday season, just days after the GASO.

**Figure 2:**
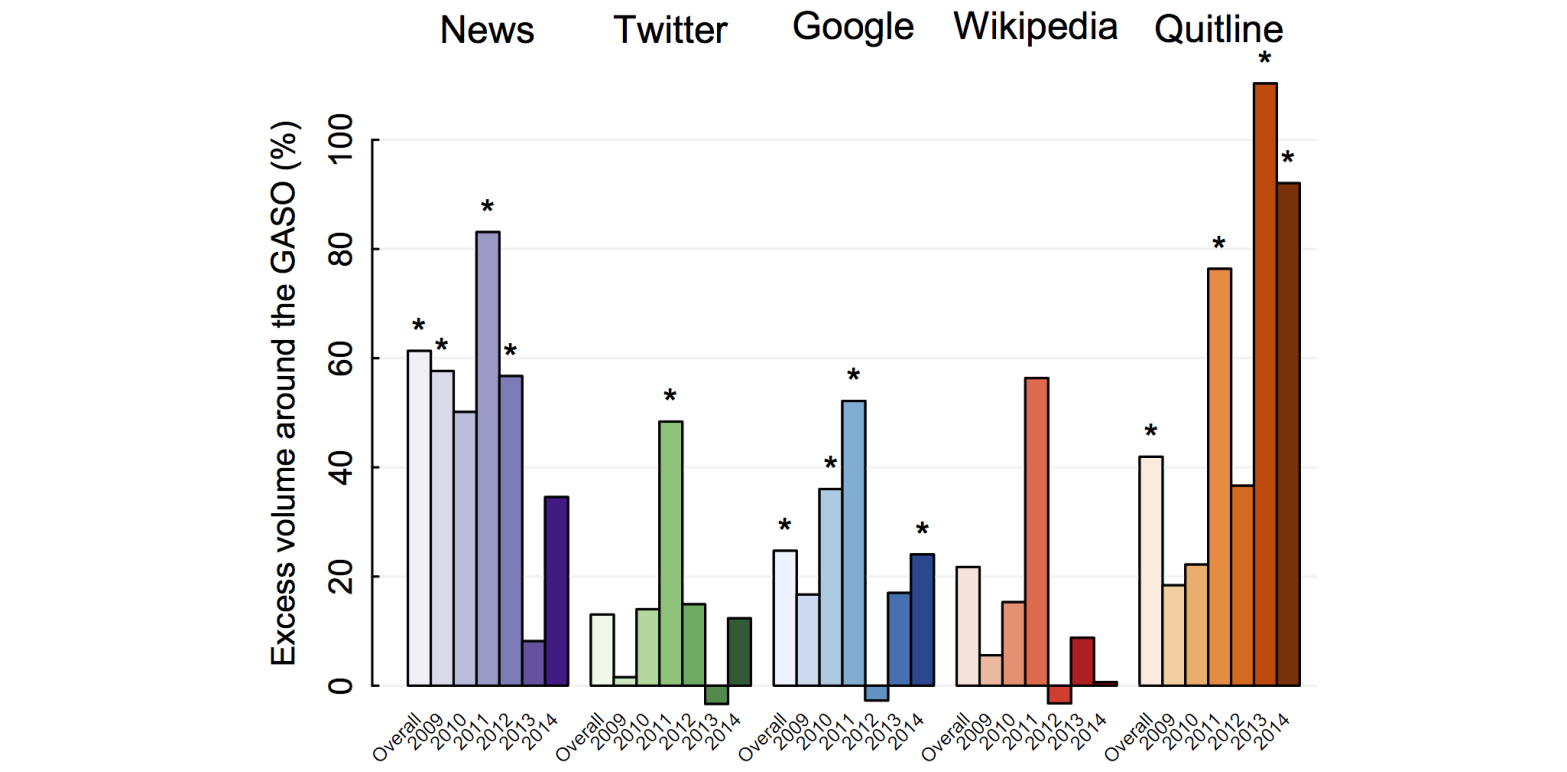
Percent increases in outcomes on the GASO compared to the counterfactual had the GASO not occurred, as detailed in the text. Asterisks indicate statistical significance (P<.05).

Statistical analysis indicated that across all years, smoking cessation news stories were 61% (95% CI 35-87) higher than would be expected if the GASO did not occur. The largest increases occurred earlier in the analyzed period and the smallest in 2013 (Figure 2). Tweets were 13% (95% CI −21 to 48) higher than would be expected, although these differences were also decreasing over the study period, ranging from an increase of 48% (95% CI 9-131) in 2011 to a nonsignificant 3% decrease (95% CI −478 to 47) in 2013. Analyses of the year-by-year relationship between news and social media coverage indicated that a 50% increase in news media was associated with an increase in tweets (28%; 95% CI −2 to 59; *P*<.06).

Google searches for cessation information were 25% higher (95% CI 10-40) across all years, with a peak increase of 52% (95% CI 26-92) in 2011, when news media and Twitter interest also peaked. Wikipedia views increased by 22% (95% CI −26 to 67) across all years but the increase was not statistically significant overall or for any one year. Call volume to quitlines increased by 42% (95% CI 19-64) across all years, with the largest increases in 2011 (76%; 95% CI 12-308), 2013 (110%; 95% CI 32-412), and 2014 (92; 95% CI 24-322). In absolute terms over the entire 5 years, this amounts to about 42,600 more Google queries, 4600 more Wikipedia visits, and 13,400 more quitline calls than would be expected had the GASO not occurred.

**Figure 3:**
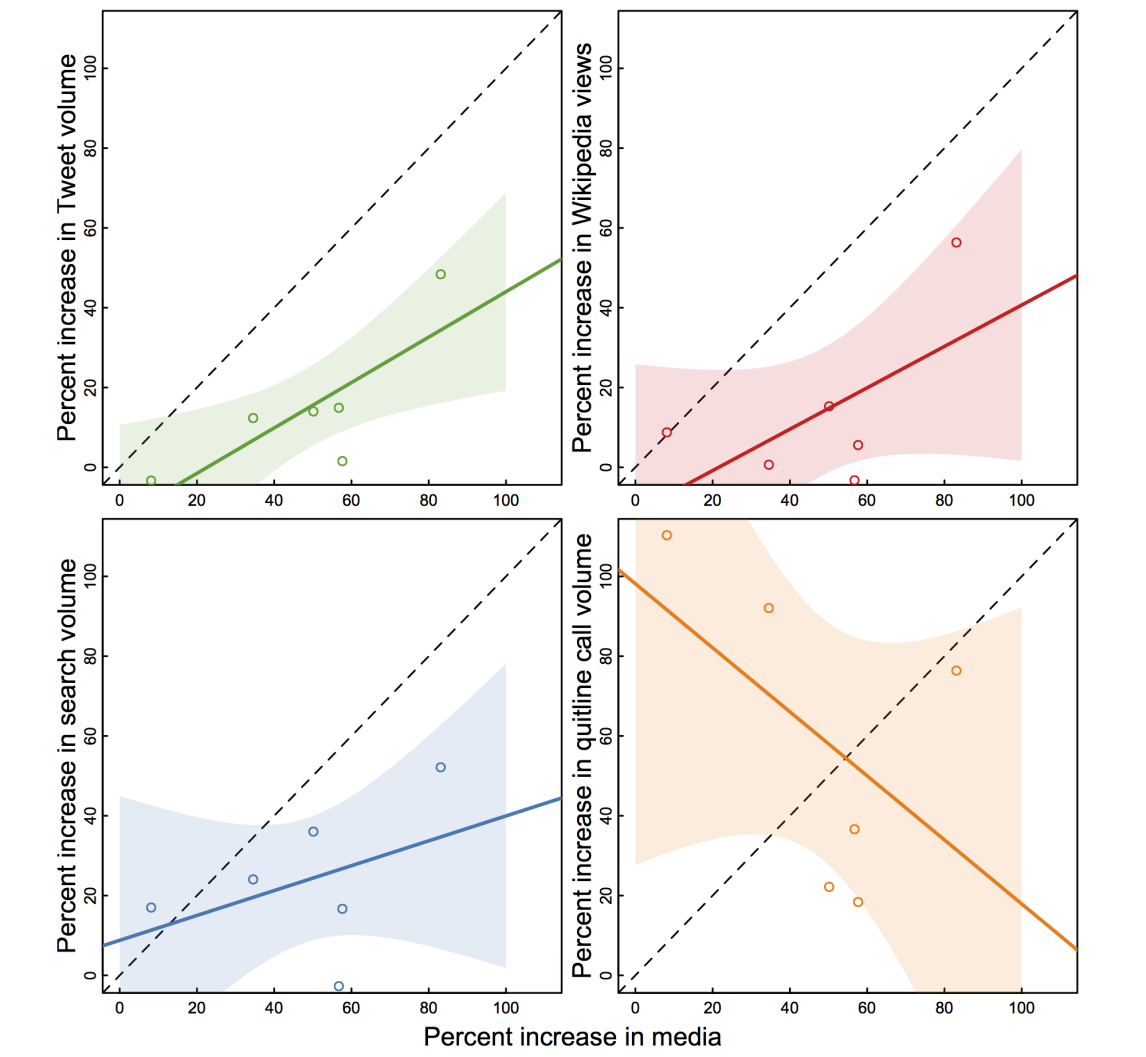
Relationships between increases in cessation-related news media and help-seeking online (Google and Wikipedia) and on quitlines. Each circle captures the co-occurring annual mean effect estimate for the outcomes as displayed on the (y) and (x) axis, with a plotted line and confidence interval as derived from a linear regression. The slope of the dashed reference line indicates a perfect one-to-one relationship between the outcomes.

Increased cessation-related news coverage was positively associated with tweets and cessation-related help seeking on Wikipedia and Google (Figure 3). Specifically, a 50% increase in cessation-related news media predicted a 12% (95% CI −22 to 46) increase in Internet cessation queries and 26% (95% CI −22 to 73) increase for Wikipedia cessation page visits, albeit neither trend was statistically significant based on a small sample of 6 years. Quitline calls were inversely related to news media, a relationship largely driven mute by the 2 most recent years when quitline call increases were greatest and news volume was lowest.

To provide a comparison for the GASO, we fitted similar models for increases around New Year’s Day, the only other period with visually significant spikes. Over all New Years (2009-2014), for example, news media coverage of cessation increased by 121% (95% CI 101-139) and Internet searches were 37% (95% CI 26-47) higher around New Year’s Day. Thus, the GASO approximates 70% of the expected increase in news media and 68% of the expected increase in cessation searches for New Year’s Day (ΔGASO divided by ΔNYD).

## Discussion

This study demonstrated a novel big data approach to rapidly, cost-effectively, and efficiently evaluate hundreds of awareness campaigns, nearly all for the first time. In doing so we found that the GASO often corresponded with increased cessation-related news coverage and cessation-related help seeking on Google, Wikipedia, and quitlines.

Our case study provided insights into how the GASO may have filtered through to the public and influenced cessation-related behavior in a way that has not been possible with traditional data. For example, we found that the GASO corresponded with increased cessation-related news coverage. Although this increase was not associated with more cessation-related tweets, it did correspond with about 61,000 more instances of cessation-related help seeking on Google, Wikipedia, and quitlines each year. These increases also compared favorably with New Year’s Day, representing about two-thirds of the effectiveness of New Year’s Day for news media and Google searches. Most critically, these novel effectiveness estimates offer suggestions for how GASO can be expanded or modified to further enhance its impact.

Because media messages need to be timed to reach receptive audiences [29], one suggestion to better ensure GASO’s cessation messages reach a more receptive audience would be to relocate these on the calendar. GASO is currently celebrated close to the holiday season when the lowest cessation-related help-seeking outcomes were observed. Add to this the recent discoveries about repeating seasonal [25] and circaseptan (day of the week) [26,27] patterns in cessation contemplations with the greatest interest during late winter and particularly on Monday, an empirically justified date of observing GASO, for example, would be the last Monday in February. With more smokers primed to consider cessation already, the GASO may very well realize larger increases apart from any specific change to its implementation.

Another potential strategy to improve effectiveness would be to attempt to increase news coverage, which, although positively related to changes in Google and Wikipedia help seeking for cessation, has been down most recently. Based on a review of prior press releases, it appears that GASO uses a repeating theme focused on smoking cessation alone. Changing themes each year to highlight novel content, such as rare health risks as with the Tips from Former Smokers Campaign [30], may engender more widespread media attention. For example, World No Tobacco Day changes themes every year and a recent analysis of that campaign showed more consistent year-to-year spikes in news coverage and correspondingly more consistent help seeking for smoking cessation on Google [12].

An enhanced strategic focus for using Twitter could also potentially increase GASO’s effect on discussions about quitting. A reliance on news media to propagate tweets is not likely to be effective given the weak relationship between news coverage and tweets. Only recently has the *American Cancer Society* addressed the GASO’s social media presence, which has included a handful of tweets announcing the GASO, and/or a few tweets with quitting tips leading up to and including the day of the GASO. Most tobacco control programs have been slow to adopt online social marketing strategies [31] and some that have engaged in these activities were quickly met with opposition or “Twitter Bombing” [32]. How best to engage awareness campaigns on social media websites, such as Twitter, continues to remain an important open question. With cessation-related tweets highest in 2011, GASO implementers might further investigate the context of that year and why tweets spiked.

The increase in online help seeking was promising, but these increases need to be concomitant with strategies to better ensure that those seeking help have an online pathway to evidence-based cessation assistance. Given the frequency with which smokers seek and find dubious cessation treatments online, such as laser therapies [33,34], a strategy to direct smokers to evidence-based cessation aids (such as information available at smokefree.gov, becomeanex.org, or cancer.org) may be beneficial for smokers attempting to quit [35]. This can be achieved in several ways such as purchasing advertisements on search engines for reliable cessation support or curating Wikipedia entries to provide reliable cessation support. For the latter, at the time we studied the general cessation Wikipedia entry, it did not include any reference to or links to proven cessation techniques, but an effort by the *Society for Nicotine and Tobacco Research* is underway to revise and update relevant Wikipedia entries. Similarly, tailoring of quitline call scripts to target GASO-motivated callers may encourage cessation by highlighting how more smokers are choosing to quit on the GASO. This could potentially increase conversions from callers contemplating to making a quit attempt via social norm mechanisms [36].

A limitation of our analyses is that (even with multiple data sources) we could not observe all the pathways by which the GASO could motivate help seeking. For example, radio discussions about cessation might motivate purchases of nicotine replacement therapies at brick-and-mortar stores, both of which we did not observe. However, given that television and radio news strongly correlate with print news in other settings [37] and the Internet is by far the most popular vehicle for health seeking, we would not expect the overall interpretation of the campaign’s effectiveness to change substantially. Still, we are actively working on identifying and adding additional data sources to our novel evaluation framework, such as Facebook [38,39]. In the same vein, our analysis focused on a single trend from each data source and did not describe subtle variations in the content of news, tweets, Google searches, etc. In subsequent evaluations, we plan to enhance our framework by diving deeper into these data, as we have done with searches for syndromic surveillance [40]. Last, the validity of our metrics is unknown because we were not able to make comparisons with surveys or other traditional data. Nonetheless, where overlap exists, our novel data sources often compare favorably with more traditional data. For example, Google searches mirror both disease outbreaks [41,42] and behavioral outcomes [43], including tobacco-related behaviors [44,45].

This study serves as an example framework for how to leverage big data for novel evaluations of awareness campaigns. As a result, the goals of awareness campaigns, like GASO’s vital aim to encourage quitting, can be better realized based on scientific data. Moreover, if GASO and related awareness campaigns are modified or enhanced based on the interpretation of our results, they can be swiftly reassessed using the same low-cost, fine-grained, and real-time big data framework.

## Abbreviations

ARIMA: autoregressive integrated moving average
GASO: Great American Smokeout
RSV: relative search volume
